# Bayesian re-analysis suggests participation rather than referral underlies the benefits of social prescribing for chronic diseases

**DOI:** 10.3389/fpubh.2026.1951236

**Published:** 2026-09-28

**Authors:** Mei Huang, Guangqing Fu, Zhen Du

**Affiliations:** 1 Department of Urology, Zigong Fourth People’s Hospital, School of Medicine, Sichuan University of Science and Engineering, Zigong, Sichuan, China; 2 School of Nursing, Southern Medical University, Guangzhou, Guangdong, China; 3 JBI Evidence-Based Nursing and Midwifery PR China, Guangzhou, Guangdong, China

**Keywords:** Bayesian analysis, chronic disease, implementation science, participation, physical activity, social prescribing

## Abstract

**Background:**

Social prescribing (SP) is increasingly implemented to address the social determinants of health in people with chronic diseases, yet uncertainty remains regarding its effectiveness and the mechanisms through which benefit is achieved. We performed a Bayesian re-analysis of randomized trials to distinguish overall treatment effects from implementation-related mechanisms.

**Methods:**

We systematically identified randomized controlled trials of SP following PRISMA 2020 guidelines. Bayesian hierarchical models were used to estimate intention-to-treat (ITT) effects on physical activity, evaluate dose–response relationships, quantify the additional benefit associated with program participation, examine patient-reported outcomes of link-worker SP, and summarize published economic evaluations.

**Results:**

10 reports involving 4,840 participants were included. SP increased the likelihood of achieving recommended physical activity levels [OR 1.31, 95% credible interval (CrI) 1.07–1.67; posterior probability of benefit = 0.991]. No consistent dose–response relationship was identified (slope −1.29 benefit units, 95% CrI − 6.17 to 3.48). In contrast, high program participation was associated with an additional 0.26 clinically meaningful benefit units (95% CrI − 0.07 to 0.63; posterior probability of benefit = 0.940). Positive effects were observed across physical activity, body weight, blood pressure, mental health, health-related quality of life, patient activation, and functional activity. Exercise-oriented SP remained cost-effective under conventional willingness-to-pay thresholds, whereas the economic value of link-worker SP depended on implementation capacity.

**Conclusion:**

SP has a high probability of improving physical activity among adults with chronic diseases. Successful participation, rather than referral alone, appears to be the principal mechanism underlying these benefits.

## 1 Introduction

Social prescribing (SP) has been suggested as a prominent strategy for addressing the social determinants of health by linking patients with chronic conditions to non-clinical, community-based resources that support physical activity, social participation, and self-management (1). In several high-income health systems, SP has evolved from a locally implemented innovation to a national endorsement of primary care over the last decade, particularly in the United Kingdom and Ireland, where link-worker services have been integrated into routine chronic disease management through dedicated policy frameworks (2, 3). The rapid expansion of these interventions reflects a growing recognition that sustainable improvements in health require interventions beyond conventional medicine. More broadly, European initiatives have increasingly positioned social prescribing as a mechanism for integrating primary care, social care, and community resources, accompanied by efforts to develop clearer standards for link-worker practice (4).

Whether SP delivers clinically meaningful health benefits, however, remains uncertain. A recent systematic review found that SP exercise referral programs produced modest improvements in physical activity, increasing weekly activity by approximately 20 min, while showing little or no effect on cardiometabolic outcomes including adiposity, blood pressure, glycemic control, or lipid profiles (5). Considering these findings, the authors concluded that there is insufficient evidence to determine whether SP promotes sustained health behavior change.

This interpretation, however informative, is inherently constrained by the estimand being addressed. Conventional intention-to-treat (ITT) analyses estimate the average policy effect of offering an intervention but do not distinguish whether variation in outcomes arises from patient adherence, intervention intensity, differential program models, or heterogeneity in treatment response (6). Consequently, the absence of large average effects does not necessarily imply the absence of clinically meaningful benefit among participants who actively engage with SP, nor does it clarify which implementation strategies are most likely to generate value (7). These mechanistic questions are particularly important for SP because its effectiveness depends on multiple behavioral and organizational processes occurring after referral rather than on a uniform therapeutic exposure.

To address these unresolved questions, we performed a Bayesian hierarchical re-analysis of randomized evidence evaluating SP for adults with chronic diseases. Rather than re-estimating only the average treatment effect, we decomposed the available evidence into complementary estimates reflecting overall effectiveness, program intensity, participant engagement, intervention model, and economic value. Specifically, we examined pooled ITT effects on physical activity, dose–response relationships between intervention intensity and clinical benefit, the additional benefit associated with high program adherence, patient-reported outcomes associated with link-worker–based SP, and cost-effectiveness across trials reporting economic evaluations. By separating referral from participation, our analysis aims to identify the mechanisms through which SP may generate clinically meaningful benefit and to inform the design of future implementation strategies.

## 2 Methods

### 2.1 Literature search and study eligibility

This study was conducted and reported in accordance with the Preferred Reporting Items for Systematic Reviews and Meta-Analyses (PRISMA) 2020 statement. We systematically searched the published literature PubMed, Embase, Web of Science, Scopus, and the ClinicalTrials from database inception to 25 June 2026 to identify randomized controlled trials evaluating social prescribing (SP) interventions. The search combined controlled vocabulary and free-text terms related to “social prescribing,” “exercise referral,” “link worker,” “community referral,” “physical activity,” and “randomized controlled trial”.

Eligible studies were randomized controlled trials evaluating healthcare-initiated referral of adults with, or at risk of, chronic diseases to non-medical, community-based programs and reporting sufficient quantitative outcome data. Non-randomized studies, protocols, and reports without extractable outcome data were excluded. Two reviewers independently screened studies, assessed eligibility, and extracted data, with disagreements resolved by consensus. Multiple reports from the same trial were linked and treated as a single study to avoid double-counting. The review was not prospectively registered; to enhance transparency, the full search strategies, data-extraction framework, and model specifications are provided in the Supplementary materials.

### 2.2 Data extraction and benefit-unit scaling

Two investigators independently extracted study characteristics, intervention duration, sample size, effect estimates, confidence intervals, adherence measures, and outcome domains from each trial. When multiple assessment time points were reported, the primary analysis used the measurement nearest program completion; the longest available follow-up was extracted separately where available. Risk of bias was independently assessed by two reviewers using the Cochrane Risk of Bias 2 tool (RoB 2 v9).

To facilitate synthesis across heterogeneous outcome domains, continuous treatment effects were transformed into clinically meaningful benefit units, in which one unit represented the established minimally important difference (MID) for the corresponding outcome. Prespecified reference values were: physical activity, 30 min/week; blood pressure, 5 mmHg; body weight, 2 kg; anxiety or depression, 2 points on HADS or equivalent scales; EQ-5D or ICECAP-A utility, 0.05; EQ-VAS or functional activity scales, 5 points; and patient activation, 4 points. Treatment effects favoring SP were coded as positive irrespective of the original reporting direction.

For trials reporting odds ratios (OR) for achieving recommended physical activity levels, analyses were performed on the natural logarithm of the OR. Physical activity risk differences were standardized relative to a prespecified 10-percentage-point reference difference. Program adherence was summarized using a composite dose proxy, defined as the proportion of prescribed sessions completed whenever available, or otherwise the reported program completion rate. Observations with missing or non-positive standard errors were excluded from model fitting using a complete-case approach.

### 2.3 Bayesian hierarchical models

All analyses were performed within a Bayesian hierarchical approach. For study *i*, the observed treatment effect *y_i_* with within-study standard error *σ_i_* was assumed to follow


$$\mathrm{yi}\sim \text{Normal}(\mathrm{\theta i},\sigma i^{2})$$

with latent study-specific effects


$$\mathrm{\theta i}\sim \text{Normal}(\mathrm{Xi\beta},\tau^{2})$$

where *Xᵢ* denotes the study-level design matrix, *β* the regression coefficients, and *τ* the between-study standard deviation.

Regression coefficients were assigned weakly informative Normal(0, 5^2^) priors. Between-study heterogeneity was modelled using a half-normal prior with a data-adaptive upper bound. Posterior inference was obtained through numerical marginalization over the heterogeneity parameter using a discrete grid approximation, followed by posterior sampling from the conditional Gaussian distribution of the regression coefficients. All analyses were performed in R version 4.5.0 (R Foundation for Statistical Computing, Vienna, Austria). No Markov chain Monte Carlo engine was used.

Four prespecified models addressed complementary estimates: (a) Overall effectiveness (ITT model). A random-effects meta-analysis estimated the pooled ITT effect on physical activity. Trials reporting binary physical activity outcomes contributed log ORs; (b) Dose–response model. A mixed-effects meta-regression evaluated the association between intervention intensity (dose proxy) and clinically meaningful benefit across outcome domains; (c) Participation model. A Bayesian meta-regression compared estimates derived from high-adherence or per-protocol analyses with corresponding ITT estimates to quantify the additional benefit associated with sustained program participation; (d) Link-worker model. A random-effects model synthesized patient-reported outcomes from trials evaluating link-worker-based SP separately from exercise-oriented interventions.

Posterior results are reported as posterior means with 95% credible intervals (CrIs) and posterior probabilities of benefit, defined as *Pr(β > 0 | data)*. This quantity represents the probability, conditional on the observed data, specified likelihood, and prior distributions, that the treatment effect favors social prescribing. Posterior probabilities were reported as continuous measures of evidential strength rather than being dichotomized using a prespecified significance threshold (8).

### 2.4 Economic synthesis

Economic outcomes were summarized using a structured descriptive approach because substantial heterogeneity existed in healthcare systems, costing perspectives, currencies, and analytic horizons across studies. Incremental cost-effectiveness ratios (ICERs), cost-effectiveness acceptability analyses, and sensitivity analyses were extracted directly from published reports and organized according to intervention model and healthcare setting. Where available, results were interpreted against current country-specific reference thresholds. These included the NICE cost-effectiveness range of £20,000–30,000 per quality-adjusted life-year (QALY) in the United Kingdom and the commonly used reference points of €20,000 and €45,000/QALY in Ireland. Sensitivity analyses describing alternative program capacity or co-payment assumptions were summarized qualitatively to illustrate how implementation characteristics influenced economic value.

## 3 Results

### 3.1 Basic characteristics

10 reports from 9 randomized controlled trials involving 4,840 participants were included (Figure 1) (2, 9–17). Study characteristics are summarized in Table 1. Risk of bias assessments are presented in Table 1 and Supplementary Figure S1. Eight trials evaluated exercise-oriented social prescribing, including exercise referral schemes, structured walking programs, Tai Chi, and community diabetes prevention interventions, whereas one evaluated a link-worker model. Four trials reported physical-activity outcomes, five reported economic evaluations, and one provided patient-reported outcomes specific to the link-worker model. Among the four trials reporting physical-activity outcomes, only Harrison et al. (12) and Murphy et al. (15) provided compatible binary estimates for achieving prespecified physical-activity thresholds and were therefore included in the pooled ITT log-odds-ratio model. Because of fundamental differences in intervention pathways, link-worker social prescribing was analyzed separately.

**Figure 1:**
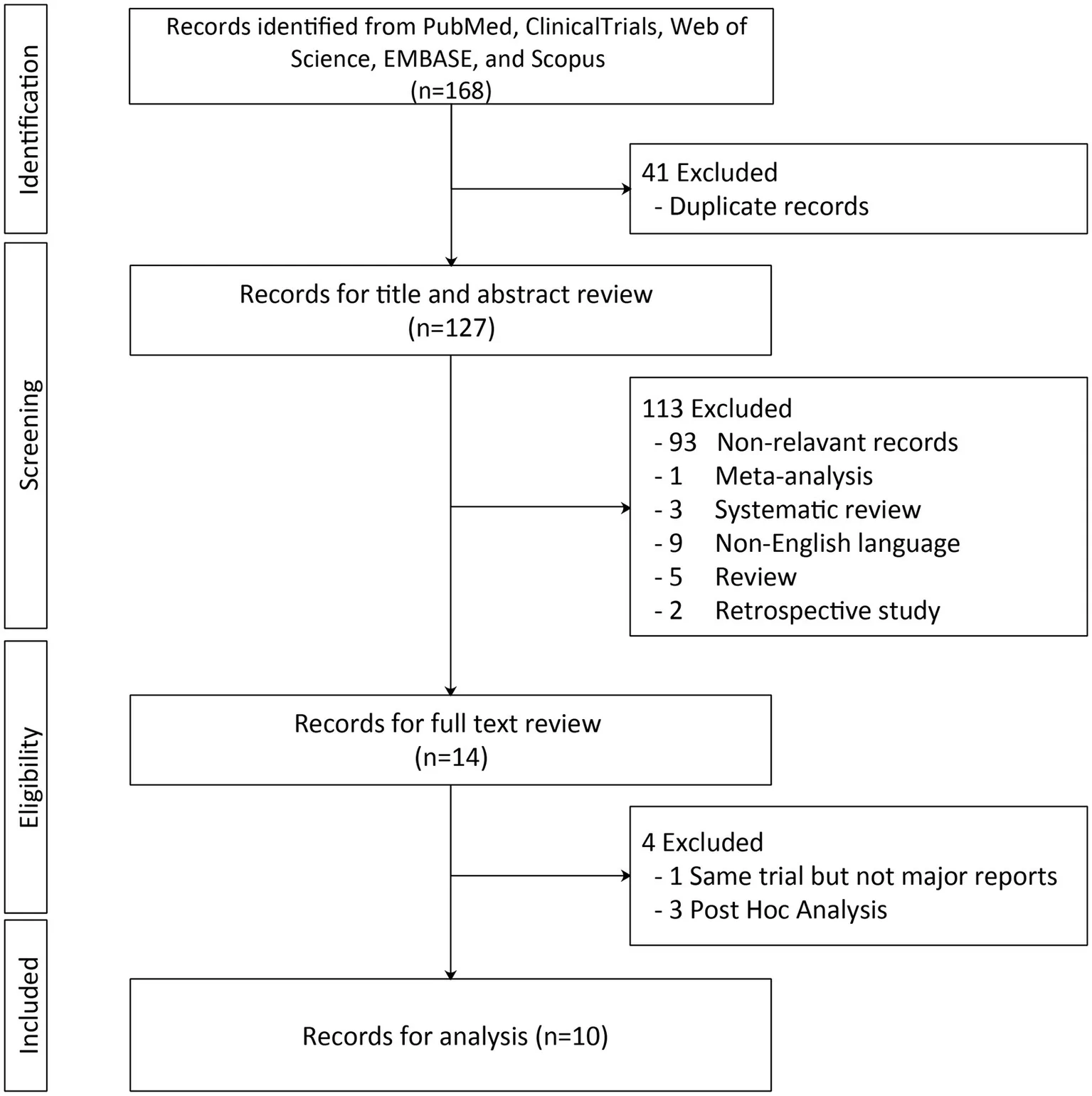
PRISMA flow diagram of study selection.

**Table 1:** Basic characteristics of included studies.

Study	*n*	Intervention type	Outcome metrics	Overall risk of bias
Gallegos-Carrillo et al. (10)	232	16-week exercise referral to Social Security Center, 3 sessions/week	Physical activity	Low
Gallegos-Carrillo et al. (9)	232	Exercise referral	Adiposity, blood pressure, mental health	Low
Gusi et al. (11)	106	GP invitation to supervised walking, strengthening and stretching, 3 × 50 min/week for 6 m	Cost effectiveness, quality of life	Some concerns
Harrison et al. (12)	545	Local-authority exercise referral plus written information	Physical activity	Low
Isaacs et al. (13)	943	Leisure-centre supervised exercise, 2–3 sessions/week for 10 weeks	Physical activity	Low
Kiely et al. (2)	240	One-month practice-based social prescribing link worker intervention	Capability wellbeing, cost effectiveness, functional activity, mental health, patient activation	Low
Ma et al. (14)	158	Group-based 24-form Tai chi training and practice for 24 weeks	Blood pressure, mental health, social support	Low
Murphy et al. (15)	2,160	16-week National Exercise Referral Scheme with motivational interviewing, goal setting and relapse prevention	Cost effectiveness, mental health, physical activity	Some concerns
Smith et al. (16)	314	Voluntary-sector diabetes prevention programme: 4 group sessions plus follow-up contacts at two, three, six, nine and 12 months alongside 5 hours of additional activities/classes	Adiposity	Low
Taylor et al. (17)	142	GP exercise prescription; 20 half-price leisure-centre sessions over 10 weeks	Adiposity, blood pressure, dose/adherence	Some concerns

### 3.2 Social prescribing improves physical activity

The Bayesian ITT analysis showed higher physical activity in the social prescribing group than in the usual-care group (Figure 2). The pooled logOR was 0.269 (95% CrI 0.063–0.512), corresponding to an OR of 1.31 (95% CrI 1.07–1.67; posterior probability of benefit = 0.991).

**Figure 2:**
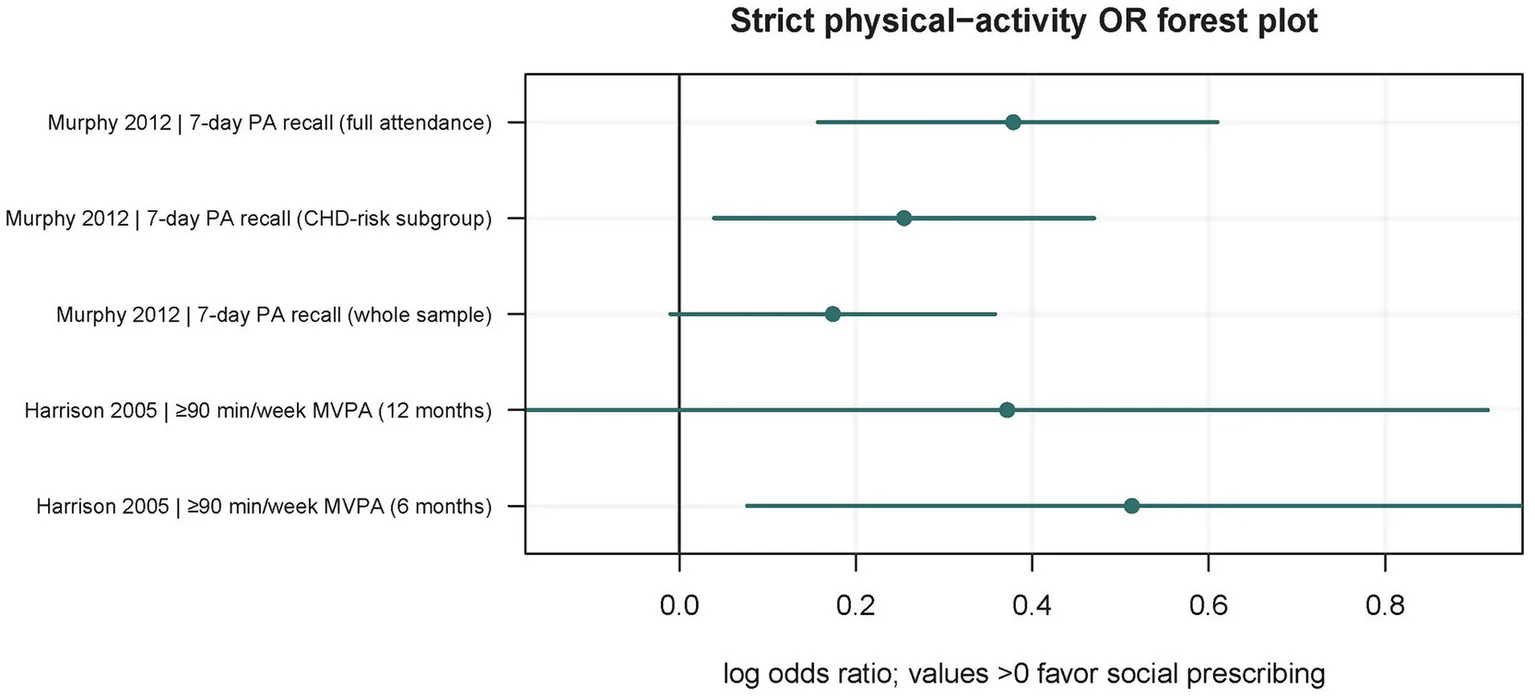
Overall effectiveness of social prescribing on physical activity. Forest plot of Bayesian random-effects ITT analysis evaluating the effect of social prescribing on achieving trial-defined physical activity levels. CrI, credible interval; ITT, intention-to-treat; OR, odds ratio; CHD, coronary heart disease; MVPA, moderate-to-vigorous physical activity; PA, physical activity.

### 3.3 Mechanisms underlying treatment benefit

After standardization into clinically meaningful benefit units, positive treatment effects were observed across multiple outcome domains, including physical activity, body weight, blood pressure, mental health, health-related quality of life, patient activation, and functional activity (Figure 3). Effect sizes varied across studies and outcome domains, with the largest estimated benefits observed for body weight and lifestyle-related outcomes. No consistent dose–response relationship was identified across studies (Figure 4). The estimated dose–response slope was −1.286 benefit units (95% CrI − 6.165 to 3.480; posterior probability of a positive slope = 0.287). High-adherence or per-protocol estimates exceeded corresponding ITT estimates across studies reporting both analyses. Compared with ITT analyses, high adherence was associated with an additional 0.260 benefit units (95% CrI − 0.069 to 0.626; posterior probability of benefit = 0.940). The link-worker model also demonstrated favorable patient-reported outcomes. The pooled benefit estimate was 0.168 benefit units (95% CrI − 0.047 to 0.414; posterior probability of benefit = 0.937). Within the Kiely trial, ITT analyses showed little change in EQ-5D-5L or HADS, whereas per-protocol analyses demonstrated greater functional activity (Frenchay Activity Index mean difference 2.1, 95% CI 0.1–4.1) and higher capability wellbeing among men (ICECAP-A mean difference 0.07, 95% CI 0.02–0.13).

**Figure 3:**
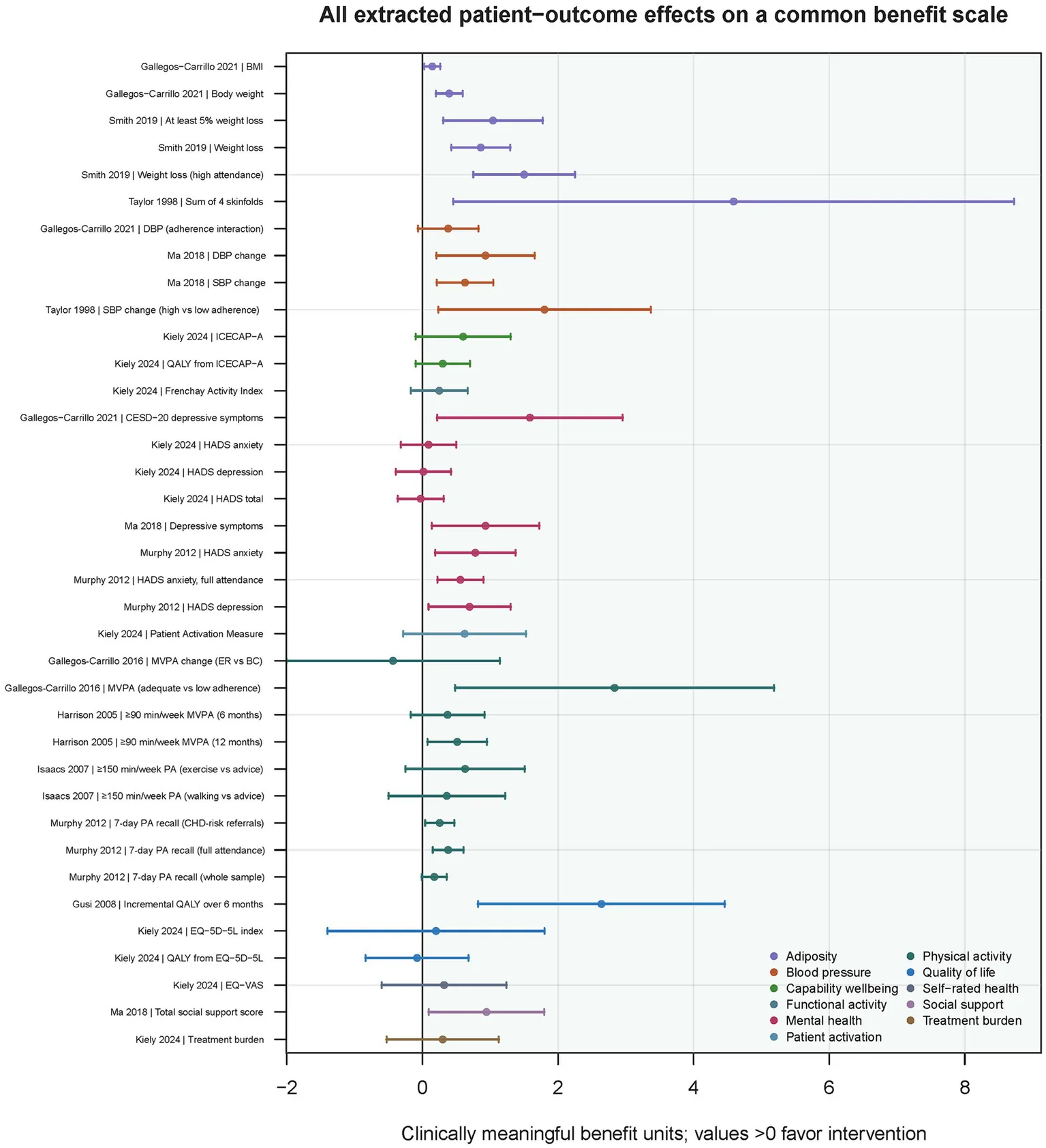
Clinical benefit across outcome domains. Forest plot showing treatment effects after standardization into clinically meaningful benefit units across outcome domains. One benefit unit corresponds to the prespecified MID for each outcome. Positive values indicate treatment benefit favoring social prescribing. BMI, body mass index; MID, minimally important difference; BC, brief counselling; CHD, coronary heart disease; DBP, diastolic blood pressure; ER, exercise referral; MVPA, moderate-to-vigorous physical activity; PA, physical activity; SBP, systolic blood pressure.

**Figure 4:**
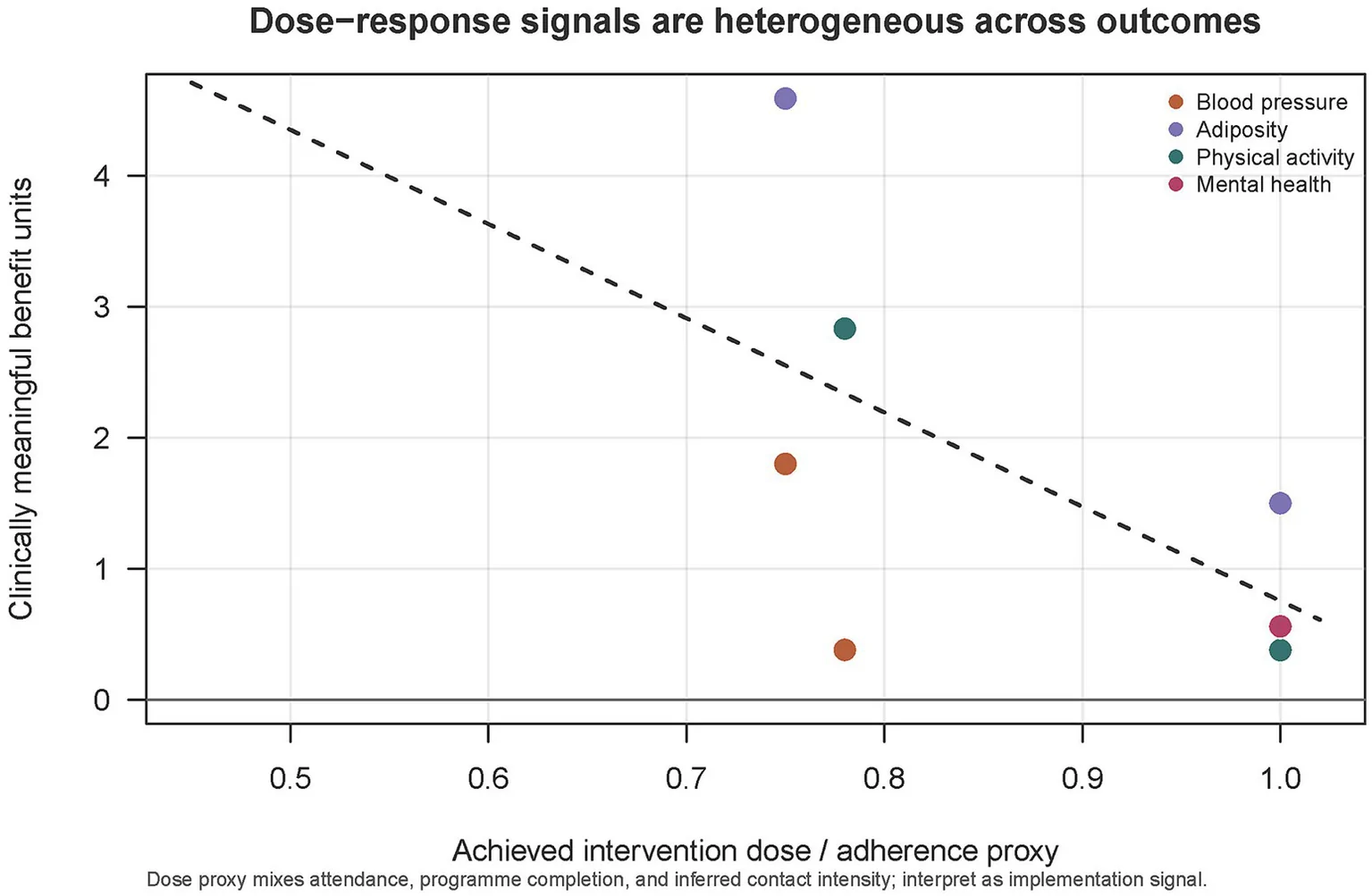
Mechanisms underlying treatment benefit. Scatter plot showing the association between achieved intervention dose or adherence proxy and clinically meaningful benefit units across outcome domains.

### 3.4 Economic value depends on implementation

Three trials reported economic evaluations. Exercise-oriented social prescribing programs demonstrated favorable cost-effectiveness, with incremental cost-effectiveness ratios ranging from €311/QALY to £12,111/QALY, remaining within the current country-specific reference thresholds. By contrast, the link-worker intervention reported a base-case ICER of €79,683/QALY. However, when implemented at full operational capacity, the probability of cost-effectiveness increased from 28.0 to 78.7%. Posterior summaries for all prespecified Bayesian models are presented in Table 2.

**Table 2:** Posterior summaries for all prespecified Bayesian models.

Model	Estimand	Posterior summary	Interpretation
Physical-activity ITT	Average log (OR)	0.269 (0.063 to 0.512)	Small but probable increase in physical activity among trials reporting OR outcomes.
Dose–response	Linear dose slope	−1.286 (−6.165 to 3.480)	Unstable because dose definitions differed across trials.
Adherence amplification	High adherence vs. referral-level contrast	0.260 (−0.069 to 0.626)	High adherence probably amplifies benefit; Pr > 0 = 0.940.
Link-worker outcomes	Mean patient-reported benefit units	0.168 (−0.047 to 0.414)	Small, uncertain, short-term signal in Kiely et al. (2); not directly comparable with exercise referral.

## 4 Discussion

This Bayesian re-analysis indicated that participation, rather than referral itself, appears to be the dominant mechanism through which social prescribing generates clinically meaningful benefit. Although social prescribing demonstrated a high posterior probability of improving physical activity at the population level, intervention intensity alone did not explain treatment heterogeneity. Instead, participants who actively engaged with and completed programs consistently experienced greater benefits across multiple health domains. This distinction shifts the interpretation of social prescribing from a referral-based intervention to an implementation-dependent behavioral strategy, with important implications for clinical practice, service evaluation, and health policy.

Our findings reinforce the conclusion of the recent systematic review that social prescribing produces modest improvements in physical activity, but extend that evidence in several important ways (5). Complementing conventional effect estimates, our Bayesian approach expressed uncertainty as the posterior probability that social prescribing was beneficial, conditional on the observed data, model assumptions, and specified priors, while separately evaluating overall effectiveness and implementation-related characteristics. The pooled ITT effect indicates that social prescribing reliably increases the likelihood of achieving recommended physical activity levels, even though the average effect size is modest. From an individual perspective, such an effect may appear small; however, from a population-health perspective, even modest behavioral changes can translate into substantial public health gains when implemented across large primary care populations, consistent with Geoffrey Rose’s prevention strategy that small improvements distributed across many individuals often produce greater population benefit than large improvements confined to a few (18).

A significant observation is the apparent discrepancy between intervention intensity and participation in the program. We found no evidence of a consistent linear dose–response relationship across studies, whereas program adherence demonstrated a high probability of amplifying treatment benefit. These findings should not be interpreted as indicating that intervention dose is unimportant. Rather, they highlight the methodological limitation that dose was defined inconsistently across trials, including program duration, attendance frequency, referral completion, and self-reported participation. Such heterogeneous metrics are unlikely to represent a common quantitative exposure. By contrast, adherence consistently captured whether participants actually received the intended intervention, irrespective of how programs were organized. Taken together, these findings suggest that participation may be a more informative implementation construct than prescribed intervention dose.

This interpretation is consistent with implementation science, which views social prescribing as a complex health-service intervention whose effectiveness depends on successful implementation rather than a fixed biological dose (19). Contemporary frameworks, including the updated Medical Research Council guidance and RE-AIM, emphasize that intervention outcomes arise through interactions between context, implementation, and participant behavior (20). Within this perspective, referral represents only the entry point to an intervention, whereas clinically meaningful benefit depends on successful engagement with community-based activities. The COM-B model offers one plausible explanation: referral creates the opportunity to participate, but sustained attendance, social support, and behavioral reinforcement are required to maintain motivation and translate opportunity into lasting behavior change. This interpretation is consistent with our finding that interventions sharing similar referral pathways may produce markedly different outcomes according to participant engagement. It is also supported by European evidence showing substantial variation in general practitioners’ awareness and use of social prescribing, suggesting that referral pathways and implementation capacity may influence whether patients successfully engage with community-based services (21). Accordingly, future social prescribing programs should prioritize strategies that enhance uptake, adherence, and long-term participation rather than increasing referral volume alone.

The observation that clinically meaningful improvements extended beyond physical activity deserves particular attention. After standardization into clinically meaningful benefit units, favorable effects were observed across body weight, blood pressure, mental health, health-related quality of life, patient activation, and functional activity. Although physical activity remains the principal outcome evaluated in most trials, these findings support the broader hypothesis that social prescribing functions as a multidimensional intervention affecting behavioral, psychological, and social wellbeing simultaneously (22, 23). The favorable mental health effects despite relatively modest improvements in physical activity suggest that social interaction, structured routine, enhanced self-efficacy, and supportive therapeutic relationships may contribute independently to program effectiveness (24).

Link-worker interventions should also be interpreted separately from exercise-oriented programs because they target broader social determinants of health through personalized community navigation (25). The modest patient-reported benefits observed in our analysis may underestimate their true effectiveness, as the only available trial was conducted during the COVID-19 pandemic with a short follow-up period. The greater responsiveness of ICECAP-A than EQ-5D and the improved cost-effectiveness under full-capacity assumptions further suggest that capability-based outcomes and implementation efficiency may better reflect the value of this model.

These findings have important implications for both practice and future research. Program success should be evaluated by participation, attendance, program completion, and sustained community engagement rather than referral volume alone, which reflects service activity more than patient benefit. Future trials should standardize reporting of intervention exposure using objective attendance measures to facilitate meaningful dose–response analyses, while economic evaluations should combine conventional health utilities with broader capability measures to capture the multidimensional aims of social prescribing. Evidence synthesis should also distinguish structured exercise interventions from link-worker models rather than treating social prescribing as a single intervention. Our study strengthens these conclusions by applying a Bayesian hierarchical framework that quantified probabilities of benefit, standardizing heterogeneous outcomes into clinically meaningful benefit units, and separating overall treatment effects from implementation mechanisms, thereby providing insights beyond those obtainable from conventional aggregate meta-analysis.

Several limitations should also be acknowledged. First, analyses were based on published aggregate data rather than individual participant data, precluding exploration of treatment-effect heterogeneity according to demographic characteristics, multimorbidity, or socioeconomic deprivation. In addition, the complete-case approach excluded estimates without usable measures of uncertainty, which may have introduced selection bias and reduced the amount of evidence available for synthesis. Several reports raised some risk-of-bias concerns, particularly regarding missing outcome data and selection of the reported result. In addition, adherence-based estimates were derived from post-randomization comparisons and should not be interpreted as causal effects. Second, clinically meaningful benefit units relied on prespecified minimally important differences, which remain debated for several outcome domains. Third, the available evidence remains limited, with most trials evaluating exercise-oriented referral programs and relatively few studies assessing broader link-worker models. Consequently, the generalizability of our findings to other forms of social prescribing, including arts-on-prescription, welfare advice, housing support, and cultural prescribing, requires confirmation in future randomized studies.

In conclusion, social prescribing is likely to improve physical activity and generate broader health benefits for adults with chronic diseases. Across studies, successful participation, rather than referral alone, consistently emerged as the key determinant of clinically meaningful benefit. These findings suggest that future programs and evaluations should focus less on referral activity and more on supporting sustained engagement with community-based interventions.

## Data Availability

Publicly available datasets were analyzed in this study. This data can be found at: all data analyzed in this study were derived from publicly available published trial reports. The data extraction table, derived analytical dataset will be provided as Supplementary material and/or deposited in a public repository upon publication.
